# Adapting culturally appropriate mental health screening tools for use among conflict-affected and other vulnerable adolescents in Nigeria

**DOI:** 10.1017/gmh.2019.8

**Published:** 2019-06-03

**Authors:** B. N. Kaiser, C. Ticao, C. Anoje, J. Minto, J. Boglosa, B. A. Kohrt

**Affiliations:** 1 Department of Anthropology, University of California San Diego, La Jolla, CA, USA; 2 Duke Global Health Institute, Duke University, Durham, NC, USA; 3 Gede Foundation, Abuja, Nigeria; 4 Catholic Relief Services, Abuja, Nigeria; 5 Department of Psychiatry and Behavioral Sciences, George Washington University, Washington, DC, USA

**Keywords:** Behavioral disorders, cultural adaptation, depression, PTSD, Nigeria

## Abstract

**Background:**

The Boko Haram insurgency has brought turmoil and instability to Nigeria, generating a large number of internally displaced people and adding to the country's 17.5 million orphans and vulnerable children. Recently, steps have been taken to improve the mental healthcare infrastructure in Nigeria, including revamping national policies and initiating training of primary care providers in mental healthcare. In order for these efforts to succeed, they require means for community-based detection and linkage to care. A major gap preventing such efforts is the shortage of culturally appropriate, valid screening tools for identifying emotional and behavioral disorders among adolescents. In particular, studies have not conducted simultaneous validation of screening tools in multiple languages, to support screening and detection efforts in linguistically diverse populations. We aim to culturally adapt screening tools for emotional and behavioral disorders for use among adolescents in Nigeria, in order to facilitate future validation studies.

**Methods:**

We used a rigorous mixed-method process to culturally adapt the Depression Self Rating Scale, Child PTSD Symptom Scale, and Disruptive Behavior Disorders Rating Scale. We employed expert translations, focus group discussions (*N* = 24), and piloting with cognitive interviewing (*N* = 24) to achieve semantic, content, technical, and criterion equivalence of screening tool items.

**Results:**

We identified and adapted items that were conceptually difficult for adolescents to understand, conceptually non-equivalent across languages, considered unacceptable to discuss, or stigmatizing. Findings regarding problematic items largely align with existing literature regarding cross-cultural adaptation.

**Conclusions:**

Culturally adapting screening tools represents a vital first step toward improving community case detection.

## Introduction

Globally, the lack of culturally appropriate mental health assessment instruments is a major barrier to screening individuals into mental health interventions and evaluating their efficacy. Simple translation of screening tools, as often used in research and practice, is inadequate and produces misleading and inaccurate conclusions (Allden *et al*., 2009). In contrast, rigorous cultural adaptation and clinical validation procedures can ensure that assessment instruments are locally appropriate and valid (Kohrt *et al*., 2011; Kaiser *et al*., 2013; Atilola, 2015). Additionally, assessment instruments that are culturally adapted function better in validation studies than tools that have not been adapted (Weobong *et al*., 2009; Ali *et al*., 2016). Across low- and middle-income countries, there are scarce validation studies for posttraumatic stress disorder (PTSD) screeners, and children and adolescents are underrepresented in validation studies for common mental disorder screeners (e.g., Ertl *et al*., 2011; Murray *et al*., 2011; Ventevogel *et al*., 2014). Adaptation and/or validation approaches have been used successfully to develop screening tools for use among adults in Nigeria (Abiodun, 1993; Omigbodun *et al*., 1996; Adewuya *et al*., 2006). Instruments that have been both culturally adapted and clinically validated do not exist for identification of children and adolescents suffering from mental, emotional, or behavioral disorders in Nigeria.

More broadly, there is a lack of research on approaches to adapting and validating tools with wide regional and linguistic applicability, for places like Nigeria that are linguistically and ethnically diverse. Globally, adaptation and validation studies focus on one language or are conducted independently in multiple languages that might be spoken in a single setting. This raises the risk that screening tools – and their resulting insight regarding referral needs or program effectiveness – might produce different results in different languages within the same setting.

Nigeria is the largest African country by population (over 180 million), with half of this number constituting children and adolescents. On top of a general environment of economic precarity, food insecurity, and other stressors has been added the violence, turmoil, and instability caused by the Boko Haram insurgency. Responsible for tens of thousands killed and millions displaced since 2009, Boko Haram was at one point considered the world's deadliest terror organization (IEP, 2015). In part due to the impact of these events, there has been an increase in adverse child and adolescent outcomes, unsurprisingly including poor mental health outcomes (Atilola, 2012). Although population-level estimates do not exist regarding child and adolescent mental disorders in Nigeria, studies from other sub-Saharan African countries yield prevalence estimates of between 13% and 20% (Cortina *et al*., 2012).

In line with World Health Organization (WHO) recommendations, Nigeria's 2013 *National Mental Health Policy* recommends task-shifting mental healthcare to non-specialist providers in primary care settings (Gureje *et al*., 2015). However, the success of these programs relies on accurate detection of children and adolescents with mental health problems in community and clinical settings. Engaging with local and community-based stakeholders to facilitate linkage of vulnerable individuals to needed mental health services is a crucial component (Adeosun *et al*., 2013; Iheanacho *et al*., 2015; Abdulmalik *et al*., 2016), but this requires persons referring children and adolescents to accurately identify who is in need. In the arena of global mental health, the use of validated screeners has been advocated as crucial to identify adults with depression who can then be referred to evidence-based psychological treatments and other services (Reynolds & Patel, 2017). This recommendation equally applies to child and adolescent mental health needs. Therefore, one key to these renewed efforts to address Nigeria's mental health needs will be the availability of locally valid screening tools with wide regional and linguistic applicability, which are needed in order to detect children and adolescents with common mental disorders and guide referral efforts from the community and primary health levels.

No studies in Nigeria have validated self-report mental health assessment tools for adolescents. Efforts have been made to assess prevalence of depression and anxiety among Nigerian adults (Adewuya *et al*., 2006, 2018; Ogunsemi *et al*., 2010; Amoran *et al*., 2012), to assess substance use and behavior disorder in adolescents (Atilola *et al*., 2016; Olagundoye *et al*., 2018; Tunde-Ayinmode *et al*., 2012), and to examine psychometric properties of mental health screening tools among children (Omigbodun *et al*., 1996; Omigbodun & Gureje, 2004; Adeniyi & Omigbodun, 2018), yet several gaps remain. These studies relied on either English versions of assessment tools or simple translation/back translation tasks, rather than rigorous cultural adaptation techniques that are needed to ensure not only semantic but also content equivalence of items prior to validation. Most studies focused on a specific population like primary care patients or university students, rather than a community-based sample. For studies among children and adolescents, parental or teacher report rather than self-report was used for all screening tools except one. In sum, although important efforts have been made to translate and examine mental health screening tools among children and adolescents in Nigeria, there remains a need for rigorous cultural adaptation as well as diagnostic validation studies (Atilola *et al*., 2015).

This study aims to contribute to Nigeria's renewed mental health policies and programs by facilitating community-level engagement and detection of mental disorders, with special attention to conflict-affected and other vulnerable adolescents. Our primary aim is to use a rigorous cultural adaptation process to develop appropriate, understandable, and easy-to-use mental health screening tools for use among Nigerian adolescents. A second aim is to demonstrate a replicable process for simultaneous adaptation of screening tools for multiple languages in the same setting. The overall goal is to support current and future interventions linking adolescents to mental healthcare, and ultimately to reduce the impact of mental health conditions among vulnerable adolescents.

## Methods

### Setting

This study, conducted in the linguistically diverse Federal Capital Territory, aims to produce tools that can be used in various parts of the country, including in the North-East, where Boko Haram's effects are felt most strongly. Abuja, the capital of Nigeria, is a planned city intentionally situated at the central point of many ethnic and religious groups. Among the dozens of ethnic groups in Nigeria, Hausa is the largest, constituting approximately 25% of the population. Hausa are largely concentrated in the north, where Boko Haram's impact is greatest. English is the national language, though more often individuals speak Pidgin, which combines English terms and grammar with terms and grammar from local West African languages. Therefore, we chose to focus on Hausa and Pidgin as the languages of focus for this study. By focusing on the two most prominent languages in Africa's largest nation by population, we anticipate the widest potential public health impact.

This project was embedded within the Sustainable Mechanisms for Improving Livelihoods and Household Empowerment (SMILE) program. The SMILE consortium is a cooperative agreement between Catholic Relief Services and the U.S. Agency for International Development, designed to scale-up care and support services for orphans and vulnerable children in four Nigerian states plus the Federal Capital Territory. The program's primary focus areas are household economic strengthening, nutrition, and HIV services. The project described here represents the first step toward incorporating a community-based mental health component into the SMILE program. This study was conducted in nine selected communities from three Area Councils in the Federal Capital Territory where the SMILE program is implemented. These represent particularly vulnerable communities selected for implementation of the SMILE program due to a high burden of socio-economic and health problems, including poverty, malnutrition, and HIV infection.

Research in Ghana showed significant variability in the mental health challenges facing different cohorts of vulnerable children (Doku, 2009). Based on experiences of the SMILE Project and local partner the Gede Foundation, we identified depression, PTSD, and behavioral disorders as the most likely mental health problems experienced among adolescents in the region.

### Instruments

The Depression Self Rating Scale (DSRS) is an 18-item self-report measure for children and adolescents (Birleson, 1981) that has been used in a range of cross-cultural contexts (Ivarsson *et al*., 1994; Denda *et al*., 2006; Panter-Brick *et al*., 2009). The Child PTSD Symptom Scale (CPSS) was developed as a child and adolescent version of the Posttraumatic Diagnostic Scale (Foa *et al*., 1997, 2001). The CPSS has 17 items that correspond to PTSD diagnostic criteria in the Diagnostic and Statistical Manual of Mental Disorders (DSM-IV; APA, 2000). The Disruptive Behavior Disorders Rating Scale (DBDRS) is a 45-item measure corresponding to DSM diagnostic criteria for attention-deficit/hyperactivity disorder (ADHD), oppositional-defiant disorder (ODD), and conduct disorder (CD) (Pelham *et al*., 1992). Due to the length of the measure, we removed the subscales for inattention and hyperactivity-impulsivity, retaining items relevant to ODD and CD. Additionally, the DBDRS was designed to be proxy-administered (by parent or teacher), so we adapted it to be a self-report measure.

### Cultural adaptation

To culturally adapt these screening tools, we applied an established, systematic process for adapting mental health screening instruments (van Ommeren *et al*., 1999), which has been adapted for use with children and adolescents (Kohrt *et al*., 2011). This process ensures correct translation not only of language, but also of full equivalence of meaning and application of tools. The cultural adaptation process involves multiple translations and focus group discussions (FGDs) in a stage-wise fashion. At each stage, translated items are considered in terms of comprehensibility, acceptability, relevance, and completeness. This aims to assess equivalence of adapted items to the original English version, in terms of semantic, content, technical, criterion, and conceptual equivalence (van Ommeren *et al*., 1999).

First, each instrument was translated into Hausa and Pidgin by local researchers not affiliated with this project. In addition to translations, they commented on equivalence of each item (comprehensibility, etc.). Each translation was then reviewed by a team of four local psychologists, who suggested alternate translations and likewise commented on equivalence. Items were then back-translated to check for completeness, and feedback from translators was used to improve each item. Finally, items were discussed by adolescents in FGDs. Rather than comparing items to the original English wording, these FGDs asked adolescents to describe each item and to comment on comprehensibility, acceptability, and relevance.

In total, 24 FGDs were conducted, stratified by gender, age (12–14/15–17), and language (Hausa/Pidgin). Each FGD had around 10 participants, except for one that had 19 due to over-recruitment. Participants were purposively selected by community volunteers affiliated with the SMILE Project. Each FGD reviewed items from one assessment instrument in one language (Hausa/Pidgin). FGDs were conducted by trilingual research assistants (RAs) gender-matched to FGD participants. RAs had undergone 2 weeks of training in project objectives, qualitative methods, and research ethics. FGDs lasted approximately 1.5–2.5 h. They were transcribed and reviewed by research coordinators and RAs to identify any responses indicating problems of equivalence, as well as to identify suggested changes to improve the items.

After completion of FGDs, items were again back-translated, reviewed, and further adjusted for clarity. Assessment instruments were then piloted using cognitive interviewing. In this method, each item is delivered to a participant, and after they respond, they are asked why they gave that response and what they understood by the item. Piloting included 25 participants, with each instrument being delivered to 9–13 individuals. Participants were purposively recruited by community volunteers as adolescents with emotional or behavior-related problems and aiming to achieve variability according to age (12–17) and gender.

The Translation Monitoring Form (van Ommeren *et al*., 1999) was used to track adjustments to item translations at each stage of data collection. FGD transcripts were reviewed to identify explicitly stated problems with items, as well as implied problems (e.g., participant giving an example that did not match the item's intended meaning, suggesting that it was not well understood). Notes regarding potential problems with items and how they were addressed were incorporated into the Translation Monitoring Form. Finally, the Form included notes regarding assessments of equivalence at each stage.

### Pilot testing

Each item was closely examined using the Translation Monitoring Form in both Hausa and Pidgin. Items were adjusted to address any challenges raised throughout data collection, with emphasis on comprehensibility and incorporating specific language suggested by FGD participants wherever possible. Efforts were made to keep items as similar as possible in Hausa and Pidgin.

Adapted versions of the screening tools were piloted in both Hausa (*N*  =  12) and Pidgin (*N*  =  12) with male and female adolescents between 12 and 17 years plus one 18-year-old (mean: 14.5). The same recruitment criteria were used as for FGDs. Cognitive interviewing was used, in which participants were asked to respond to each item and then describe their decision-making process. Specifically, participants were asked ‘Why did you give that response?’ and ‘How did you understand that question?’ The purpose of the cognitive interviewing task was to have participants verbalize their interpretation of items in order to identify any items that seemed to be interpreted differently than intended. Following pilot testing, additional adjustments were made to items as needed to improve comprehensibility.

Quantitative pilot data were analyzed for descriptive statistics, internal consistency using Cronbach's *α*, and item-total correlations. Additionally, sum scores were calculated using mean imputation, and *t* tests were performed to compare group means between Pidgin and Hausa versions of the screening tools.

All study procedures were approved by the Federal Capital Territory Health Research Ethics Committee (approval number FHREC 2016/01/44/22/06/16). Before data collection, community chiefs provided *loco parentis* consent on behalf of adolescents, and all adolescents assented before participating.

## Results

### Adaptation results

Several types of changes were made to the screening tools to address problems with comprehensibility, conceptual non-equivalence, acceptability, lack of specificity, and stigma.

#### Facilitating comprehensibility

In order to improve comprehensibility, we made several changes to all screening tool items. First, we changed items to be framed as questions rather than statements. We also added the specific time period to the start of each item. For example, each DSRS question now begins with ‘In the past week.’ Additional changes for comprehensibility included identifying culturally specific terms and idioms to best capture each item; for example, the PTSD item ‘unable to have strong feelings’ (CPSS-11) was worded as ‘having a dry heart’ (*zuciyanka ya bushe*) because adolescents suggested this as the clearest way to capture the concept in Hausa.

#### Conceptually difficult items

Despite efforts to improve comprehensibility, several items remained conceptually difficult for adolescents to understand. For example, it was difficult for adolescents to grasp the concepts of ‘looking forward’ to the future (DSRS-1) or making future plans (CPSS-12). Similarly, CPSS-3 (re-experiencing) was difficult for adolescents to differentiate from fearing that the same traumatic event might happen again. Two items on the DBDRS scale addressed similar concepts with nuanced distinctions: angry or resentful (item 7) and spiteful or vindictive (item 8). Every effort was made to identify the best local terms to capture these concepts. Nevertheless, they often remained difficult particularly for younger adolescents to understand due to the relatively high-level concepts.

A related challenge is that – particularly when attempting to describe relatively abstract concepts – items sometimes became relatively long. FGD participants sometimes commented that items became complex and somewhat difficult to follow. They recommended delivering items slowly and carefully to aid comprehension. Similarly, one item (DBDRS-10: initiating fights) includes clarification that it does not relate to fights with one's brother or sister. However, in Hausa there are no single terms for brother and sister but only for older brother, younger sister, etc. This item therefore became substantially longer in Hausa than in Pidgin.

#### Conceptually different meanings

Several items were interpreted differently from the original English item. For example, the original translation of DSRS-15 (feeling lonely) was feeling that it is ‘only you in this life.’ FGD participants interpreted this item as referring to being rich and having the ability to live by yourself. The item was therefore changed to feeling you don't have anyone in life (Pidgin: *you no get anybody for this life*). Similarly, in cognitive interviewing, CPSS-16 (overly cautious) was sometimes interpreted as assessing whether adolescents displayed an *appropriate* degree of caution, for example checking whether people are around you. However, most cognitive interviewing participants understood the item as referring to being overly cautious.

An additional challenge regarding conceptual equivalence related to items that were not specific to emotional or behavioral distress. For example, cognitive interviewing participants reported not sleeping through the night (DSRS-2) due to waking up for chores or studying. Additionally, two items were adjusted to account for common experiences of hunger or lack of food. DSRS-6 (tummy aches) was adjusted to clarify ‘not caused by hunger or sickness,’ and DSRS-8 (enjoying food) was clarified by adding ‘when food is available.’ These adjustments were made in order to most closely capture the specific meaning intended by the original English items, rather than unrelated experiences such as hunger.

#### Acceptability and stigma

There were several items that were described during FGDs as potentially being unacceptable or uncomfortable for adolescents to endorse. For example, participants reported that adolescents might not want to talk about a negative home environment (DSRS-5: running away), won't want to be reminded of a traumatic event (many CPSS items), and won't want to be seen as bad children, thieves, liars, disobedient, or disrespectful to adults (several DBDRS items). Because it is not feasible to adjust these items to make them more acceptable, efforts were made to focus on developing rapport and encouraging adolescents to be comfortable reporting their experiences. Additionally, adolescents reported that in the local culture, you do not tell your dreams to people (DSRS-14, CPSS-2: bad dreams). However, the research team felt that adolescents will be comfortable reporting *that* they had bad dreams as long as they do not have to report their content.

An additional challenge with acceptability related to items that were considered stigmatizing. For example, the initial translation of DSRS-10 (feeling that life is not worth living) was phrased as ‘wanting to die.’ However, FGD participants reported that it is taboo to talk about suicide or dying. The item was therefore adjusted to more closely mirror the original item, feeling that life is not worth living. Additionally, discussion of sex was initially described as unacceptable (DBDRS-15: forced sex), so the item was phrased as ‘forcing someone to sleep with you.’ However, the item caused confusion, with some adolescents interpreting it as forcing one's siblings to share a bed for company. Additionally, adolescents seemed comfortable using the term rape in FGDs. It was therefore decided to use the wording ‘forcing someone to sleep with you’ and only clarifying with the term for rape when adolescents did not understand.

### Pilot test results

Mean age of pilot test participants was 14.5 years. Just over half (58%) were male. Scores did not differ significantly between Pidgin and Hausa versions (Table 1). Pilot test results showed generally strong internal consistency for each screening tool (Cronbach's *α*  =  0.61–0.79), except for the Pidgin version of the DBDRS, which had acceptable internal consistency (*α*  =  0.52), likely due to low endorsement of CD items.

**Table 1.** Mean imputed scores on culturally adapted screening tools (*N*  =  24)

	Total possible score	Pidgin median, IQR (*N* = 12)^a^	Hausa median, IQR (*N* = 12)	Wilcoxon rank test
Depression (DSRS)	36	12.0 (9.0)	6.0 (6.0)	*S* = 145.5, *p* = 0.06
PTSD (CPSS)	51	14.4 (10.0)	14.9 (12.5)	*S* = 148.0, *p* = 0.91
Disruptive behavior (DBDRS)	39	8.8 (3.5)	6.5 (4.5)	*S* = 115.0, *p* = 0.05

IQR, Interquartile range.

a DSRS Pidgin: *N*  =  10.

The pilot test results also suggested several potentially problematic items (Tables 2–4). Due to the very small size of the pilot test (*N*  =  10–12 for each screening tool), these preliminary results are limited for drawing inferences. At the same time, several of these items were also identified as problematic during qualitative data collection, suggesting that they might indeed require further adjustment. For example, CPSS-3 (re-experiencing) had a very low item-total correlation in both Pidgin and Hausa, suggesting either that the concept was not well-understood or that it does not fit local experiences of PTSD. Similarly, ‘looking forward to things’ (DSRS-1) had been reported as conceptually difficult and showed low item-total correlations on both Pidgin and Hausa screening tools.

**Table 2.** Pilot test of Depression Self-Rating Scale among Nigerian adolescents (*N*  =  22)

		Pidgin (*N* = 10, *α* = 0.77)		Hausa (*N* = 12, *α* = 0.61)	
Item	Original English	Mean (s.d.)	Item-total correlation	Mean (s.d.)	Item-total correlation
1^a^	I look forward to things as much as I used to	1.00 (0.5)	−0.12	0.50 (0.7)	−0.25
2^a^	I sleep very well	0.80 (0.4)	0.55	0.42 (0.7)	−0.14
3	I feel like crying	0.70 (0.7)	0.04	0.25 (0.5)	0.53
4^a^	I like to go out to play	1.00 (0.5)	0.25	0.75 (0.5)	0.50
5	I feel like running away	0.30 (0.5)	0.33	0.08 (0.3)	0.61
6	I get tummy aches	0.50 (0.5)	0.09	0.33 (0.5)	−0.24
7^a^	I have lots of energy	0.80 (0.4)	0.39	0.50 (0.5)	0.58
8^a^	I enjoy my food	0.33 (0.5)	0.84	0.33 (0.5)	0.42
9^a^	I can stick up for myself	1.00 (0.5)	−0.28	0.50 (0.7)	0.23
10	I think life isn't worth living	0.56 (0.5)	0.62	0.50 (0.7)	−0.06
11^a^	I am good at the things I do	0.40 (0.5)	0.82	0.25 (0.5)	0.53
12^a^	I enjoy the things I do as much as I used to	0.60 (0.5)	0.43	0.42 (0.5)	0.34
13^a^	I like talking with my family	0.40 (0.5)	0.31	0.25 (0.5)	0.27
14	I have bad dreams	0.70 (0.5)	0.24	0.33 (0.5)	0.24
15	I feel very lonely	0.40 (0.5)	0.62	0.17 (0.4)	0.64
16^a^	I am easily cheered up	0.80 (0.4)	0.53	1.25 (0.6)	−0.02
17	I feel so sad I can hardly stand it	0.50 (0.5)	0.45	0.50 (0.5)	0.46
18	I feel very bored	0.80 (0.4)	0.53	0.42 (0.5)	0.34

a Positively-worded items were reverse-scored.

**Table 3.** Pilot test of Child PTSD Symptom Scale among Nigerian adolescents (*N*  =  24)

		Pidgin (*N* = 12, *α* = 0.72)		Hausa (*N* = 12, *α* = 0.79)	
Item	Original English	Mean (s.d.)	Item-total correlation	Mean (s.d.)	Item-total correlation
1	Having upsetting thoughts or images about the event that came into your head when you didn't want them to	0.83 (0.8)	0.47	1.17 (1.1)	0.62
2	Having bad dreams or nightmares	0.67 (0.8)	0.17	0.42 (0.7)	0.33
3	Acting or feeling as if the event was happening again (hearing something or seeing a picture about it and feeling as if I am there again)	0.42 (0.5)	0.08	0.75 (0.9)	0.08
4	Feeling upset when you think about it or hear about the event (for example, feeling scared, angry, sad, guilty, etc.)	1.00 (0.7)	0.60	1.27 (1.0)	0.73
5	Having feelings in your body when you think about or hear about the event (for example, breaking out into a sweat, heart beating fast)	0.58 (0.7)	0.17	1.08 (1.0)	0.57
6	Trying not to think about, talk about, or have feelings about the event	1.33 (1.1)	0.58	1.00 (1.0)	0.39
7	Trying to avoid activities, people, or places that remind you of the traumatic event	1.25 (1.2)	0.37	1.50 (1.3)	0.60
8	Not being able to remember an important part of the upsetting event	0.80 (1.0)	0.08	0.83 (0.9)	0.63
9	Having much less interest in doing things you used to do	1.00 (1.0)	0.09	0.92 (1.2)	0.55
10^a^	Not feeling close to people around you	0.75 (1.1)	0.04	0.75 (1.1)	−0.27
11	Not being able to have strong feelings (for example, being unable to cry or unable to feel happy)	0.33 (0.5)	0.17	0.33 (0.5)	0.55
12	Feeling as if your future plans or hopes will not come true (for example, you will not have a job or getting married or having kids)	0.25 (0.9)	0.17	0.08 (0.3)	0.34
13	Having trouble falling or staying asleep	1.17 (1.2)	0.32	0.67 (1.1)	0.61
14	Feeling irritable or having fits of anger	1.42 (1.1)	0.56	0.45 (0.5)	−0.03
15	Having trouble concentrating (for example, losing track of a story on the television, forgetting what you read, not paying attention in class)	1.00 (1.0)	0.55	0.92 (0.8)	0.50
16	Being overly careful (for example, checking to see who is around you and what is around you)	1.09 (1.0)	0.54	1.82 (1.3)	0.20
17	Being jumpy or easily startled (for example, when someone walks up behind you)	0.75 (0.5)	0.35	0.58 (0.8)	0.37

a Following adaptation, item was asked in the positive (‘do you feel close to people?') and was reverse-scored.

**Table 4.** Pilot test of Disruptive Behavior Disorders Rating Scale among Nigerian adolescents (*N*  =  24)

		Pidgin (*N* = 12, *α* = 0.52)		Hausa (*N* = 12, *α* = 0.79)	
Item	Original English	Mean (s.d.)	Item-total correlation	Mean (s.d.)	Item-total correlation
1	Loses temper	0.75 (0.5)	0.08	0.75 (0.6)	0.27
2	Argues with adults	0.75 (0.5)	0.26	0.42 (0.7)	0.35
3	Actively defies or refuses to comply with adults' requests or rules	0.50 (0.5)	0.08	0.58 (0.5)	0.21
4	Deliberately annoys people	0.82 (1.0)	−0.09	0.42 (0.9)	0.64
5	Blames others for his/her mistakes or misbehavior	0.50 (0.5)	0.05	0.25 (0.5)	0.61
6	Is touchy or easily annoyed by others	0.92 (0.3)	0.22	0.67 (0.5)	0.39
7	Is angry and resentful	0.83 (0.4)	0.50	0.67 (0.5)	0.21
8	Is spiteful or vindictive	0.42 (0.5)	0.16	0.33 (0.5)	0.56
9	Often bullied, threatened, or intimidated others	36%	−0.08	50%	0.32
10	Often initiated physical fights	25%	0.67	8%	0.74
11	Used a weapon that can cause serious physical harm to others (e.g., a bat, brick, broken bottle, knife, or gun)	17%	0.10	0%	–
12	Has been physically cruel to people	25%	0.19	17%	0.18
13	Has been physically cruel to animals	58%	−0.25	17%	0.47
14	Has stolen while confronting a victim (e.g., mugging, purse snatching, extortion, armed robbery)	8%	0.12	0%	–
15	Has forced someone into sexual activity	0%	–	0%	–
16	Has deliberately engaged in fire setting with the intention of causing serious damage	0%	–	0%	–
17	Has deliberately destroyed others' property (other than by fire setting)	8%	0.13	0%	–
18	Has broken into someone else's house, building, or car	0%	–	0%	–
19	Often lies to obtain goods or favors or to avoid obligations (i.e. ‘cons' others)	50%	0.67	42%	0.49
20	Has stolen items of nontrivial value without confronting a victim (e.g., shoplifting, but without breaking and entering)	36%	0.25	8%	0.74
21	Often stays out at night despite parental prohibitions	58%	0.01	67%	0.35
22	Has run away from home overnight at least twice while living in parent's home, foster care, or group home	8%	0.58	8%	−0.04
23	Is often truant from school	17%	0.45	33%	0.61

Other problematic items were not so conceptually complex but might be problematic for other reasons. For example, CPSS-10, which was altered to the positively worded ‘feeling close to people around you’ and reverse-scored, had either a low (Pidgin) or negative (Hausa) item-total correlation. It might be that having a single positively-worded item on the screening tool led to it being misunderstood by a large portion of the sample. In contrast, the DSRS has approximately equal numbers of positively and negatively worded items, and there was no consistent pattern in terms of one type of item performing better or worse. DSRS-6 (tummy aches) might have been problematic if it were interpreted as non-specific to mental illness; however, it did not have high levels of endorsement as might be expected if that were the case. At the same time, it had low item-total correlations, making it difficult to determine whether including the clarification of ‘not caused by hunger or sickness’ helped make the item equivalent to the original English item.

## Discussion

This is the first study to conduct rigorous cultural adaptation of screening tools simultaneously in two languages. It is also the first study to culturally adapt screening tools for detection of mental and behavioral disorders among adolescents in Nigeria. Developing easy-to-use, comprehensible, and locally appropriate mental health screening tools is a vital first step in leveraging community-based detection of mental disorders.

### Adaptation challenges

Challenges encountered and changes required to culturally adapt the screening tools are consistent with other cultural adaptation literature. For example, several items were interpreted in FGDs as non-specific to mental illness, such as tummy aches, not enjoying food, or trouble sleeping. Previous studies have likewise found that such items require specific adaptations to differentiate them from experiences of diarrheal disease, hunger, or other stressors (Kohrt *et al*., 2007, 2011, 2016; Hanlon *et al*., 2008; Kaiser *et al*., 2013; Haroz *et al*., 2017; Mazzuca *et al*., 2019). While some of these items appeared to function well following adaptation (e.g., not enjoying food), other items (e.g., tummy aches) were endorsed in a different pattern than other items, suggesting that they might continue to be endorsed outside the experience of emotional distress.

Some items were problematic in both qualitative and quantitative findings, including CPSS-3 (re-experiencing) and DSRS-1 (looking forward to things), suggesting either that these concepts were not well-understood or that they do not fit local experiences of PTSD and depression. Carrion *et al*. (2002) have proposed that re-experiencing is less common in youth, and Kar *et al*. (2007) found it to be one of the least endorsed PTSD symptoms in a sample of Indian youth. Hanlon *et al*. (2008) found the concept of ‘looking forward to’ not to translate effectively in Amharic, and Dyregrov & Yule (2006) have suggested that foreshortened future is common in trauma-exposed children and adolescents. Among earthquake-affected children in California, feelings of foreshortened future did not distinguish between those with and without PTSD on the CPSS (Foa *et al*., 2001). Additionally, we found it difficult to capture subtle differences of meaning in adapting items (e.g., angry *v*. resentful, spiteful *v*. vindictive), which other researchers have reported in cultural adaptation studies (Hanlon *et al*., 2008).

Some items that have been identified as unacceptable or stigmatizing in other settings were problematic in only one language in our pilot study (e.g., crying, suicidal ideation; Miller *et al*., 2006; Ventevogel *et al*., 2007; Kumar *et al*., 2015; Housen *et al*., 2018). It might be that final translations were more acceptable for one language than another, which might reflect difficulties balancing comprehensibility and acceptability in adapting items. Significantly, several authors have found suicidal ideation to be considered unacceptable during qualitative research that informs cultural adaptation, but that participants nevertheless endorse such experiences in piloting and validation studies (Hanlon *et al*., 2008; Kaiser *et al*., 2013; Kohrt *et al*., 2016; Housen *et al*., 2018; Mazzuca *et al*., 2019). We similarly found suicidal ideation to be considered unacceptable to ask about in some FGDs but that such experiences were endorsed by participants. It might be that experiences that are highly stigmatized like suicidal behavior are reported as unacceptable by some groups but as important signs of distress to others, particularly vulnerable groups (Kohrt *et al*., 2016; Mazzuca *et al*., 2019). It is therefore important to elicit perspectives from multiple groups – including particularly vulnerable groups – to understand how best to incorporate questions about significant forms of distress. In this study, we found that suicidal ideation had a low item-total correlation in pilot data for only one language, suggesting that more work might be needed to adapt this item. Table 1

Additionally, we found that the single positive valence item on the CPSS performed poorly; other studies have similarly found that positive items can be difficult to interpret, particularly when valence changes within a screener (Weobong *et al*., 2009; Kam & Zhou, 2015; Kohrt *et al*., 2016). Finally, others have found that editing each item to be framed as a question and to remind respondents of the time period aids with technical equivalence when adapting items from self-report to interviewer administered (Hanlon *et al*., 2008; Weobong *et al*., 2009; Kohrt *et al*., 2011, 2016; Housen *et al*., 2018).

In contrast, some of our findings do not agree with literature in other settings. For example, participants emphasized acceptability problems with the DBDRS items related to being seen as a bad child, disobedient, or disrespectful to adults. These findings might reflect the local eco-cultural context, specifically cultural norms regarding children and adolescents’ behaviors (Super & Harkness, 1986; Atilola, 2015; Burkey *et al*., 2016). For example, arguing with or disobeying an adult (symptoms of ODD) is considered strongly taboo, as children ‘are to be seen, not heard.’ In contrast, behaviors like fighting, lying, and stealing – particularly among peers – (symptoms of CD) might represent a means of expressing distress that are more socially sanctioned in Nigeria. Additionally, in the study region, there is an increase in general violence and children and adolescents’ engagement in gangs. In most Western settings, a lenient family environment might make symptoms of ODD more culturally acceptable, while well-functioning legal systems generate strong deterrents to CD behaviors. In contrast, settings like Nigeria are marked by strong social sanctions against ODD behaviors within the family, whereas the normalization of violent or criminal behavior combined with a weak legal system might allow CD behaviors to be more prominent. At the same time, we found that both ODD and CD items were problematic in piloting. Further research is needed to characterize acceptability and frequency of behavioral disorder items.

### Adaptation across languages

Significantly, we found that some items differed by language. For example, one item (DBDRS-10) became substantially longer in Hausa than in Pidgin due to the existence of many distinct terms to reference siblings in Hausa. Additionally, one item (CPSS-11) was adapted by using a relevant idiom of distress in Hausa, whereas an equivalent idiom of distress was not identified for Pidgin. This item performed better in Hausa than Pidgin, as evidenced by item-total correlations. Because idioms of distress can be a particularly promising way to identify and communicate about mental distress, efforts should be made to incorporate them into screening tools where possible (Kohrt *et al*., 2011, 2016; Rasmussen *et al*., 2015; Weaver & Kaiser, 2015; Fabian *et al*., 2018). However, this raises challenges when attempting to adapt screening tools across multiple languages and cultural groups, which might make use of different concepts including idioms of distress to make sense of and communicate mental distress. Additionally, care should always be taken so that idioms of distress are not reduced to psychiatric categories or symptoms (Nichter, 2010; Kohrt *et al*., 2014; Kaiser *et al*., 2015). Differences we identified by language are of particular concern in studies like this that aim to simultaneously adapt screening tools that can function equivalently across multiple languages. This study makes an important contribution in this area, but further research is needed to develop best practices for screening tool adaptation for linguistically diverse populations like Nigeria.

In future, we recommend training community health extension workers and community volunteers, like those trained by SMILE and other existing programs, to use and interpret the screening tools described here. These trainings should include processes for referral to specific care providers for those individuals identified as potentially experiencing a mental disorder. The development of service directories and referral pathways should occur alongside efforts to train individuals in community-based detection. Additionally, we recommend that primary care providers be trained in the use of these screening tools as part of efforts to scale-up the mhGAP program nationally. Finally, dissemination efforts should be paired with research and actively seeking feedback from those implementing the tools.

### Limitations

Several challenges and limitations might have influenced study findings. For example, the research team decided to culturally adapt screening tools for use in Pidgin and Hausa in order to prepare tools that would be useful not only in Abuja but also nearby regions. However, during FGDs and cognitive interview piloting, we found that in some communities, many adolescents did not speak Hausa. Although community volunteers were asked to recruit both Pidgin and Hausa speakers, several FGDs had to be switched to Pidgin upon realizing the language barrier. This might be because, although Hausa is a prominent language in the broader region, within the Federal Capital Territory, it is not the dominant language or ethnic group. Second, female FGD participants tended to be less talkative than males. These limitations might mean that cultural adaptation was ultimately better achieved for Pidgin screening tools and/or for males. It might be the case that some items showed different response patterns compared to the rest of their respective scale because they do not match local experiences of depression, PTSD, and behavior disorder and should be removed from the screening tool. However, due to the small sample size, we are limited in terms of being able to draw statistical inferences. Finally, we chose to focus on self-report measures because our measures aim to be of greatest use in humanitarian settings where, for example, teacher report is less feasible. However, future studies should aim to validate parent or teacher report versions of the instruments as well, particularly for externalizing disorders. Similarly, although we removed the ADHD sub-scale of the DBDRS due to its length, future studies should validate this or other ADHD scales in order to capture this important aspect of behavioral disorder in Nigeria.

## Conclusion

Our study demonstrates the benefits and challenges of undergoing simultaneous adaptation of screening tools in multiple languages. We identified and attempted to resolve problems with comprehensibility, acceptability, and non-equivalence across languages. For the most part, findings regarding items that were difficult to adapt match existing literature, with the exception of patterns of endorsement for items related to ODD and CD. The resulting culturally adapted screening tools should be locally validated and used to inform community-based detection and referral efforts, in order to link adolescents to mental health care, especially for conflict-affected and other vulnerable adolescents in Nigeria.

## Supplementary material

For supplementary material accompanying this paper visit http://dx.doi.org/10.1017/gmh.2019.8. click here to view supplementary material
